# Supplementary material for: Rapid turnover of life-cycle-related genes in the brown algae
Source: Genome Biol. 2019 Feb 14;20:35. doi: 10.1186/s13059-019-1630-6 (PMC6374913; doi:10.1186/s13059-019-1630-6)
Supplement: Supplementary file 3 — Protein alignments, corresponding Gblocks html file and CDS sequences for the lineage conserved gametophyte-biased genes. (ZIP 2109 kb) [file 13059_2019_1630_MOESM3_ESM.zip › Con_GA_alignments/Ec-09_004140.aa_ali.fasta-gb.htm]

Ec-09\_004140.aa.fasta.trans.aa\_ali.fasta


## Gblocks 0.91b Results

Processed file: **Ec-09\_004140.aa.fasta.trans.aa\_ali.fasta**  
Number of sequences: **2**  
Alignment assumed to be: **Protein**  
New number of positions: **381** (selected positions are underlined in blue)

```
                         10        20        30        40        50        60
                 =========+=========+=========+=========+=========+=========+
Ec-09_004140     ---------------------------MSEVAINALTVDLTRLLSS------AKGRQSTE
MSTRG.57309.1    RIFRSRTCARRLNPLWPDSRPRCPKANMSEVAIDALRVDLTRLLLSARSCGPAKGRPETD
                                            ###################      ########


                         70        80        90       100       110       120
                 =========+=========+=========+=========+=========+=========+
Ec-09_004140     DLAETTSLFRQCKQNS-GVKENSGVKENSETVARDIGGEISTTPGSPVGSTLTRKTPLPD
MSTRG.57309.1    QIAKTTSLFRRCKENSQGDAGNSVSEAEGRSVVDGLVDVAAATPATTT--AVSRRSPLPD
                 ################ ###############                     #######


                        130       140       150       160       170       180
                 =========+=========+=========+=========+=========+=========+
Ec-09_004140     EVDSCCYAYAPLRDQKPTTVAAAAAAAEEEIKARPPGRKVPPASPSHGDVDRRAYTYADG
MSTRG.57309.1    EVGSCCYTYAPLVDE-----PKPAATVPSKVESRSPVKGARPASPSHEDVDRRVYTYADG
                 ##############         #####################################


                        190       200       210       220       230       240
                 =========+=========+=========+=========+=========+=========+
Ec-09_004140     FKPRRRDQQPPAAASSSFSQNG--ASRKWRDR-RSNRRTSGPHFPGRQHQHHQHQQQHQQ
MSTRG.57309.1    FKLKRRRRRQPNAACFTFSRTGEFHSRRWHDRSASSRCVPSPNSPGTPPQ----------
                 ######################            ################          


                        250       260       270       280       290       300
                 =========+=========+=========+=========+=========+=========+
Ec-09_004140     QQQDQQQHQQQQDQLRQDPDAQVNHEAPSRDGEGGTDTARTVRREDAQPRDAVLVGEAWS
MSTRG.57309.1    ----QLAHKKSKNGVFSGDENSCAA---------TVPIAMTDGREEGPPRVPMRVGEAWS
                                                       ######################


                        310       320       330       340       350       360
                 =========+=========+=========+=========+=========+=========+
Ec-09_004140     ENAAGGIEGSIGRERDRASVGDAGSQ---RPSAESRRDSSELHEFLYREGRSREDRRRAK
MSTRG.57309.1    ENAVLEDKAWQGQGAPMVCSKATGSQPLEASMPPSRRDSSDLHEFLHREGRNREDRLKAK
                 ############                      ##########################


                        370       380       390       400       410       420
                 =========+=========+=========+=========+=========+=========+
Ec-09_004140     QAERDRELTFRPRLATTKYWAAATARRRRRS------RADDFGLATERGRDGAGAAAEDA
MSTRG.57309.1    QAERDRELTFQPRLATTKYWAAVARRRRRRAACMPSREEEQAEVGSTITADIAGYQEKHA
                 ##############################                    ##########


                        430       440       450       460       470       480
                 =========+=========+=========+=========+=========+=========+
Ec-09_004140     RAGGGV-----EGVPARFEMLYEDSKAQLLRSQEDRVKIPAACTFRPQLISYRPTSRGRG
MSTRG.57309.1    HGGAALAAMRNSGAQGRFEMLYQDSKAKLLRAQGDKLRIPAACTFQPRLSSRQSPTRERG
                 ###         ################################################


                        490       500       510       520       530       540
                 =========+=========+=========+=========+=========+=========+
Ec-09_004140     RHVARKATAAAAAASPEDADMGRETYVFDDSGELFDESVNDPHARNERGDSDGLDNGNDD
MSTRG.57309.1    RQLVRDTSAGVPEEPPGRANAVGDAYVLAGSAGCTSEHVGGRREDKHGG----DDGGNDD
                 #######################################               ######


                        550       560       570       580       590       600
                 =========+=========+=========+=========+=========+=========+
Ec-09_004140     DSQAWVNSLVFGDNDEDDGHVDNSRSSGANDNGDNNSNNTKRTEEGRGQEQHGDRPRRFD
MSTRG.57309.1    DSQAWVNSLVFDGMDGSKQTSNNSRNAACDDD----------------------------
                 ###############################                             


                        610       620       630       640       650       660
                 =========+=========+=========+=========+=========+=========+
Ec-09_004140     RHGSRLRTPRAGLRGENGRGYGEIRSSSSSNGGLSAVDGPSEEGSTGNRRPGGCNRRDYH
MSTRG.57309.1    ------------------------------------------------------------
                                                                             


                        670       680       690       700       710       720
                 =========+=========+=========+=========+=========+=========+
Ec-09_004140     RRSRSLPSRSRGVTRSGLSEGAGGRTGAVGTPSYAERLYRGHDEQQQVWARRRQERARLE
MSTRG.57309.1    ------------------------------------------------------------
                                                                             


                        730       740       750       760       770       780
                 =========+=========+=========+=========+=========+=========+
Ec-09_004140     LKGCTFRPTLDQNSQNLGRAGRTYLGEGQNGGARRRSSSAVRWKRTTPQEEEKEEEEASG
MSTRG.57309.1    ------------------------------------------------VEEEEEEEE---
                                                                             


                        790       800       810       820       830       840
                 =========+=========+=========+=========+=========+=========+
Ec-09_004140     REGGMAAAAAAAAAHERLYQAAEHVRARRREEVAERRKAETVGCTFQPEVNHAPAASCAA
MSTRG.57309.1    ------------------------------------------------------------
                                                                             


                        850       860       870       880       890       900
                 =========+=========+=========+=========+=========+=========+
Ec-09_004140     AFQGRGRSTYSGGTAKRNGGDRRGVRSGSVNSNNGCRNTSRDNGSSVFDRLVAAGEEAKQ
MSTRG.57309.1    ------------------------------------------------------------
                                                                             


                        910       920       930       940       950       960
                 =========+=========+=========+=========+=========+=========+
Ec-09_004140     RKAMLGKMTPPGCTFRPQVNVHGNGKRPPGHASNSAGSTQNGGGTFAAQSQPHPQHPPPS
MSTRG.57309.1    ------------------------------------------------------------
                                                                             


                        970       980       990      1000      1010      1020
                 =========+=========+=========+=========+=========+=========+
Ec-09_004140     SYGGAPRSEENVSNFPPPPPPQTGSYHSNPGNDGYGKDGNRPPPPRRPSAGAPGGSGDWA
MSTRG.57309.1    ------------------------------------------------------------
                                                                             


                       1030      1040      1050      1060      1070      1080
                 =========+=========+=========+=========+=========+=========+
Ec-09_004140     VTQKGRRSDEGSVAGISDTAAHHAADKNFLRELAAAASTAAASAADAVPVAEVDEEGARI
MSTRG.57309.1    ------------------------------------------------------------
                                                                             


                       1090      1100      1110      1120      1130      1140
                 =========+=========+=========+=========+=========+=========+
Ec-09_004140     PYRESRFSPRRRRRKQGGGYDGNGNDGHGGGESGRESPACERLFELGHRQQRKRNDSPRD
MSTRG.57309.1    ------------------------------------------------------------
                                                                             


                       1150      1160      1170      1180      1190
                 =========+=========+=========+=========+=========+=
Ec-09_004140     TPQSQRERDELEELKLCTFSPVLFTKAYRRPRPRLATGAPSNRMEATGKRG
MSTRG.57309.1    --------------------------------------------------X
```

```
Parameters used
Minimum Number Of Sequences For A Conserved Position: 2
Minimum Number Of Sequences For A Flanking Position: 2
Maximum Number Of Contiguous Nonconserved Positions: 8
Minimum Length Of A Block: 10
Allowed Gap Positions: None
Use Similarity Matrices: Yes
```

```
Flank positions of the 11 selected block(s)
Flanks: [28  46]  [53  76]  [78  92]  [114  134]  [144  202]  [215  230]  [279  312]  [335  390]  [411  423]  [433  519]  [535  571]  

New number of positions in Ec-09_004140.aa.fasta.trans.aa_ali.fasta-gb:  381  (31% of the original 1191 positions)
```
